# Effects of Nonthermal Plasma on Morphology, Genetics and Physiology of Seeds: A Review

**DOI:** 10.3390/plants9121736

**Published:** 2020-12-09

**Authors:** Pia Starič, Katarina Vogel-Mikuš, Miran Mozetič, Ita Junkar

**Affiliations:** 1 Jožef Stefan Institute, Jamova cesta 39, 1000 Ljubljana, Slovenia; katarina.vogelmikus@bf.uni-lj.si (K.V.-M.); miran.mozetic@ijs.si (M.M.); ita.junkar@ijs.si (I.J.); 2 Biotechnical Faculty, University of Ljubljana, Jamnikarjeva ulica 101, 1000 Ljubljana, Slovenia

**Keywords:** cold plasma, seeds, seed priming, plants, germination, yield, gene expression, (de)methylation, growth, ROS, phytohormones, MDA, antioxidant enzymes

## Abstract

Nonthermal plasma (NTP), or cold plasma, has shown many advantages in the agriculture sector as it enables removal of pesticides and contaminants from the seed surface, increases shelf life of crops, improves germination and resistance to abiotic stress. Recent studies show that plasma treatment indeed offers unique and environmentally friendly processing of different seeds, such as wheat, beans, corn, soybeans, barley, peanuts, rice and *Arabidopsis thaliana*, which could reduce the use of agricultural chemicals and has a high potential in ecological farming. This review covers the main concepts and underlying principles of plasma treatment techniques and their interaction with seeds. Different plasma generation methods and setups are presented and the influence of plasma treatment on DNA damage, gene expression, enzymatic activity, morphological and chemical changes, germination and resistance to stress, is explained. Important plasma treatment parameters and interactions of plasma species with the seed surface are presented and critically discussed in correlation with recent advances in this field. Although plasma agriculture is a relatively new field of research, and the complex mechanisms of interactions are not fully understood, it holds great promise for the future. This overview aims to present the advantages and limitations of different nonthermal plasma setups and discuss their possible future applications.

## 1. Introduction

Plasma is the fourth fundamental state of matter [1]. It is a mixture of electrons, positively charged ions, radicals, gas atoms, molecules (in excited or basic state) and photons from a range of energies including ultraviolet (UV) and vacuum ultraviolet (VUV) radiation [2,3]. This state is typical of a number of natural phenomena on Earth, such as cloud-to-ground and cloud-to-cloud lightning, the aurora borealis and fire. In space, plasma is the main component of matter, including the solar corona and solar winds. It is found in the tails of comets, in the interstellar and intergalactic media and in the accretion disks around black holes. Under laboratory conditions, it is obtained by supplying energy to a neutral gas, causing the excitation of gaseous molecules and atoms and at least partial dissociation and ionization. The energy can be supplied by heating or exposure to an electromagnetic field [4,5].

Plasma can be categorized according to its thermal equilibrium or nonequilibrium. When generated by heating the gas to a sufficiently high temperature to produce ionized gas it is termed thermal plasma. The constituents of thermal plasma are in thermodynamic equilibrium, possibly at temperatures well above 1000 °C. Thermal plasmas are generated primarily by different gaseous discharges, including direct current (DC), alternate current (AC), radio frequency (RF) and microwave (MW) discharge. A wide range of DC and RF plasma sources with power levels ranging from a few W to several MW levels [6] are available.

In a nonequilibrium plasma or nonthermal plasma (NTP) the excitation, dissociation or ionization of molecules is more effective than increasing the average kinetic energy by heating, resulting in lower temperatures of the heavy particles in comparison to the electron temperature. While the electron temperature is usually of the order of 10,000 K, the temperature of heavy particles is often close to the room temperature. NTP plasma or cold plasma is more suitable for the treatment of thermally unstable biological samples than thermal plasma because the heating of the samples is minimal. The heating can be further suppressed when it comes to short-term NTP exposures, for example, in pulsed modes [2,3,7].

NTP has a wide range of biological applications, from decontamination and sterilization of surfaces [8], food [9], treatment of medical implants for biocompatibility [10], improved wound healing [11], seed germination [12,13,14] and resistance to certain abiotic stresses [15]. NTP technology is becoming increasingly popular in agriculture, in particular for seed treatments. Studies have shown a positive effect on seed sterilization, presenting an elegant solution for the reduction of the amount of chemical pesticides used, to diminish the high burden on the environment [16] and high risk for human health [17]. NTP has also been shown to improve seed germination and viability, which in turn could help to avoid the use of chemical substances for seed priming and increase the yield of various important crops [18,19,20,21].

**Figure 1:**
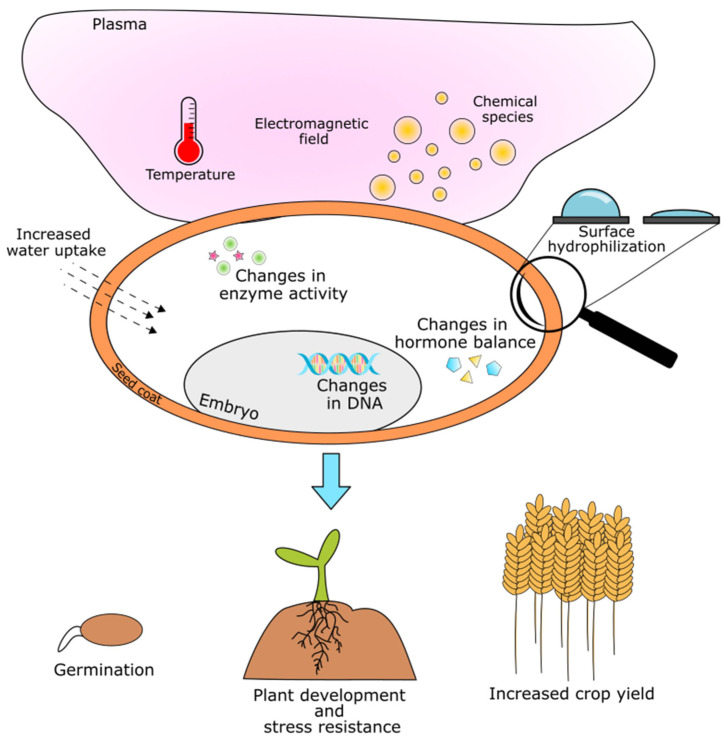
A schematic representation of nonthermal plasma (NTP) effects on seeds.

Interaction of gaseous plasma with the seed surface is schematically presented in Figure 1. Reactive chemical species, the electromagnetic field and the temperature of plasma exert some influences on the seed material. In most cases the seed surface becomes more hydrophilic as the competition between the functionalization of the surface molecules and etching of the surface takes place. Hydrophilicity is mainly related to the removal of waxy structures from the surface of seeds by plasma etching. This can affect the seed morphology, as changes of nanostructures on the seed surface may be observed. The interaction of reactive chemical species in plasma also changes surface chemistry (functionalization of the surface), which may improve surface wettability, water uptake and, consequentially, initiate complex signaling pathways in seeds. Changes in DNA, enzymatic activity and hormone balance have also been detected, depending on the plasma treatment conditions. Summarily plasma-induced changes can affect germination, later growth and development of plants, their resistance to abiotic stress and yield. However, plasma and discharge parameters should be optimized to obtain desired seed responses regarding water uptake, sprouting, growth, etc.

In this review, we focus on physiological and morphological aspects of the effects of NTP on seeds and plants. The present knowledge enables the prediction of possible influences of NTP on seeds in terms of germination and growth, stress resistance, DNA change, transfer of traits to the next generation, enzyme activity, phytohormones and, to some extent, optimization by the choice of the plasma treatment conditions [21,22,23,24]. There is already some evidence of the positive effects of NTP on seeds, but it is still unclear what mechanisms are behind the improved seed germination and viability. It is important to emphasize that different research groups use different experimental setups and parameter settings, as well as different types of seeds, which all affect the results of plasma-seed interactions. All results presented in this review and summarized in Table 1 are not necessarily applicable to all types of seeds. However, a basic overview is given.

### 1.1. Nonthermal Plasma Generation Methods

**Figure 2:**
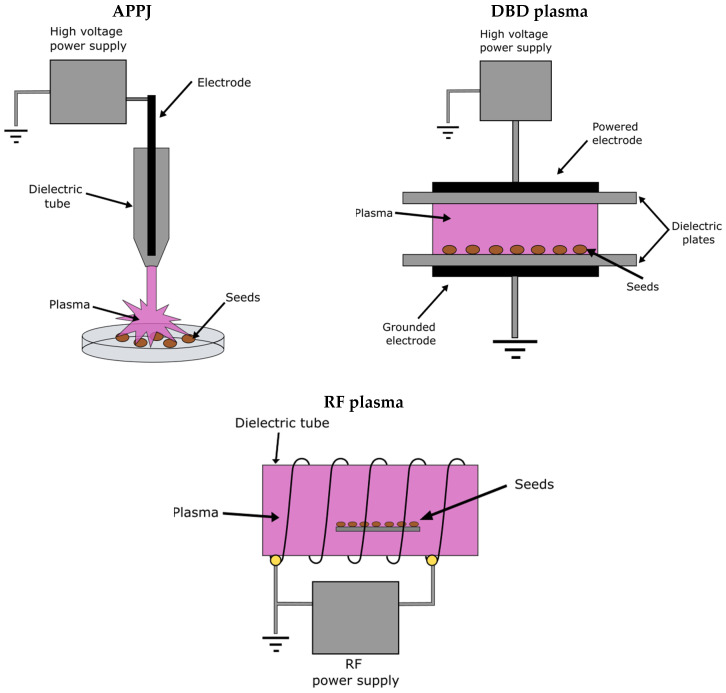
Positioning of seeds in different plasma reactors.

Although authors used a variety of experimental setups, the most common methods for NTP generation used for seed treatment are presented in Figure 2 and are based on atmospheric pressure plasma jet discharge (APPJ) [34,45,46], dielectric barrier discharge (DBD) [13,18,19,20,21,23,24,25,28,32,33,36,39,40,42,44] and radio-frequency discharge (RF) [13,22,26,27,29,37,38,43,47,48,49,50]. The type of discharge depends on the frequency of the power source (AC, DC), the ambient gas pressure (low and atmospheric pressure) and the exact shape of the electrodes [51]. The most commonly used methods for seed treatment are the DBD and RF discharges, which are described below in more detail.

A plasma reactor with DBD consists of two electrodes covered with a solid insulating material (glass, plastic, ceramic, etc.). Such dielectric coating prevents the arc discharge that would cause the generation of thermal plasma. The gas temperature usually does not rise significantly because the DBD plasma runs in streamers of very short time duration, typically in the microsecond range. DBD is generated by applying alternating (AC) high-voltage with a frequency from 50 Hz up to about 100 kHz [52].

RF plasma is normally generated by an RF power supply at 13.56 MHz. The frequency is high enough to provide a sustaining continuous plasma, so no streamers are observed. We can further classify the RF plasma by the shape of the electrodes or the antenna. In capacitively-coupled plasma (CCP), RF power is applied between two electrodes and plasma is generated in the gap between. In inductively-coupled plasma (ICP), an antenna has the shape of a coil to which the RF voltage is applied. The plasma is then generated inside the coil. RF set-up can be used to generate a relatively large volume of rather high-density plasma, with minimal heating under low-pressure conditions [51].

The exact mechanisms that contribute to seed priming by using NTP are not yet well understood. Experiments on seeds to observe the effects of direct (glow mode) and indirect (afterglow mode) plasma treatment could help to reveal the mechanisms by which NTP interactions with seeds affect surface properties and seed germination [51].

#### Direct and Indirect Effects of Nonthermal Plasma Treatment

**Figure 3:**
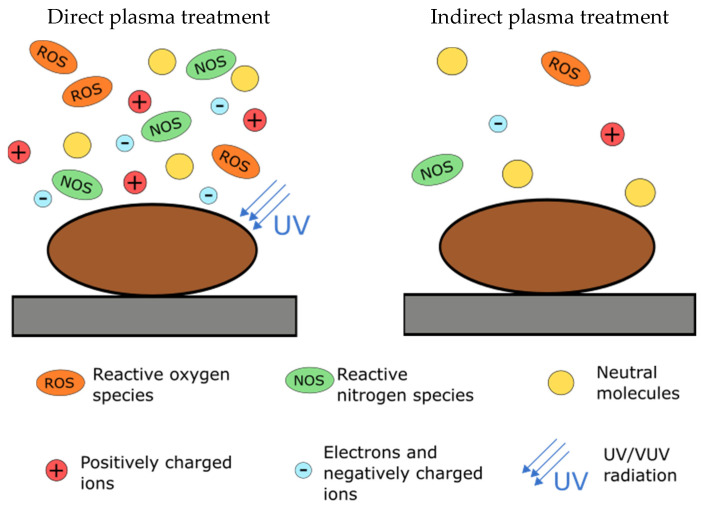
Schematic representation of particles occurring in direct and indirect plasma treatment region. Direct plasma treatment region is a combination of positively and negatively charged particles, neutral chemical species, ROS, NOS and UV radiation. In indirect plasma treatment, however, only lower, less aggressive concentrations of reactive chemical species are present.

The seeds or other biological samples can be exposed to different modes of NTP depending on the plasma system. The treatment can be performed in the so-called direct or glow mode, or indirect (afterglow) mode. Surfaces that are exposed to the area of discharge (direct plasma treatment) are subjected to UV and/or VUV radiation and the plasma particles (ions, electrons and different excited atomic and molecular species). During indirect treatment, however, the samples are exposed to nonequilibrium gas outside of the area of the glowing plasma region but close enough to benefit from interaction with long-lived radicals. Samples, in this case, are not exposed to the radiation and are subjected to lower, less aggressive concentrations of reactive chemical species with lower energy, brought from the discharge area by the gas flow. These samples are thus exposed only to longer-lived or recombined species, which are the result of secondary reactions. These species have marginal kinetic energy and lower potential energy compared to plasma species. Thermal heating of samples in the afterglow mode is marginal but can still functionalize the seed surface [53]. Indirect plasma treatment is usually weaker, and longer times of sample exposure are needed to achieve similar results as in direct plasma treatment [54]. Moreover, the etching of surfaces in this region is significantly lower. Interactions of plasma species in the direct and indirect modes are schematically presented in Figure 3.

## 2. Nonthermal Plasma Effects on Seeds

### 2.1. DNA Methylation and Demethylation and DNA Damage

The use of NTP technology on seeds can affect their different physiological and morphological levels. Plasma generation causes formation of different reactive species such as oxygen (ROS) and nitrogen (RNS) reactive species, and UV/VUV radiation that can have some effect on DNA of the embryo. Although there are no reports on DNA damage in seeds, such as double-strand breaks (DSBs) or formations of micronuclei, some experiments showed that NTP treatment caused changes in DNA (de)methylation and gene expression. Experiments conducted by Leduc et al. [55] showed that NTP treatment could potentially cause DSBs and fragmentation of plasmid DNA in case of isolated DNA in phosphate-buffered saline (PBS buffer) or water. It is important to emphasize that the fragmentation of isolated (naked) DNA by NTP does not mean that the treatment of seeds with NTP could result in similar effects, even at the same NTP parameters. Another study conducted by Lazovic et al. [56] showed that atmospheric pressure plasma induced single-strand breaks and base-damage of DNA in the human primary fibroblast cells, depending on the plasma treatment time and power. However, it should be noted that embryonic cells in seeds have cell walls that to a certain degree protect the cells from abiotic stresses and that the seed coat (testa) also acts as a protective barrier. This is the reason that short-term exposure to NTP should not harm the DNA in seeds at least not to the degree of the total DNA fragmentation [55]. Here, it should be stressed that the plasma particles do not penetrate deep into the solid matter (surface treatment technique, where only the top few nanometers of surface are modified), while the radiation can penetrate rather deeply, depending on the photon energy. For example, the UV radiation of the germicidal range of wavelengths (about 230 –280 nm) can penetrate the bacterial cell wall, causing DNA damage [57].

Kyzek et al. [36] showed that NTP treatment of pea seeds did not cause significant DNA damage. When pea seeds were treated with zeocin, that has toxic effects and causes severe DNA damage, seeds pretreated with NTP showed a significantly lower level of DNA damage. Although a low level of DNA damage induced by NTP cannot be excluded, it is possible that NTP triggers DNA repair mechanisms that repair damaged segments of DNA and protect against additional DNA harming agents. Further studies are, however, needed to prove the upregulation of DNA repair mechanisms in seeds treated with NTP.

In recent studies, Tomeková et al. [35], examined the effects of longer exposure times of pea seeds to cold plasma. They used air, nitrogen, oxygen and combinations of O_2_ and N_2_ in different ratios, as a feed gas for plasma treatment. The study analyzed the composition of the gaseous product of the plasma with FTIR spectroscopy and correlated the spectroscopy results with analysis of DNA damage in pea seeds treated with cold plasma at different conditions. The results indicated that ambient air had the least damaging effects on pea seed DNA. The DNA was more damaged with increasing concentration of nitrogen and exposure time. It was estimated that the optimal combination of the plasma chemical composition and water vapor in ambient air had a positive effect on pea seed germination. However, further research is necessary to confirm this. At the same time, it is necessary to separately investigate the influence of chemically reactive gaseous species and plasma radiation. As mentioned above, the reactive species are unlikely to reach the DNA, whereas the radiation rather easily penetrates into the organic material. The effects may be separated by placing a VUV-transparent window between the glowing plasma and organic matter, which is a practice when studying the effects on polymers [58].

In another study Nakano et al. [27] conducted a microarray analysis on *Arabidopsis thaliana* seeds. The seeds treated with oxygen NTP at a pressure of 20–80 Pa and power of RF generator 60 W displayed an increase in DNA methylation. The NTP treatment also enhanced the growth and germination of seeds. The analysis of microarray results showed a decrease in expression of *AT2G30620*, a gene encoding histone H1.2, which is involved in repression of DNA transcription. Its downregulation implies changes in the chromatin structure. On the other hand, the studies have shown an increase in the RNA-directed DNA methylation 4 gene (*RDM4*) encoding proteins connected with DNA methylation. This observation indicates that NTP treatment may influence the epigenetics of seeds to a certain extent. Treatment with argon NTP exhibited different expression patterns for the before-mentioned genes, with the expression levels of *AT2G30620* remaining the same, but the expression of *RDM4* was lower compared to the oxygen NTP treatment at a low pressure of 20 Pa, indicating different effects of different NTP feed gases. One possible explanation is in the difference in VUV radiation. Low-pressure oxygen plasma is not a significant source of radiation; usually, only the O-line at about 130 nm is observed, whereas atmospheric pressure plasma sustained in pure argon is an extensive source of VUV continuum arising from Ar_2_^*^ dimers [59]. Zhang et al. [24] used an atmospheric pressure discharge and found that argon NTP can reduce methylation and increase gene expression of adenosine triphosphate (ATP) synthase, target of rapamycin (TOR) and growth-regulating factor (GRF) genes in soybeans involved in the biosynthesis of energy-rich molecules and biomass production, and regulate the growth and development of seedlings. A higher concentration of ATP promotes germination and growth of sprouts, which has been shown in germination and growth experiments with soybeans. All these results suggest that NTP has an effect on the epigenetics of seed embryos, but the mechanisms behind its activation are still unknown. Unfortunately, many authors do not report on peculiarities of discharges, in particular the gas purity, which may be the decisive parameter governing the radiation and chemical reactivity of NTPs [60].

### 2.2. Gene Expression and Protein Synthesis

The effects of NTP described above show that NTP could actually affect seed gene expression. This implies possible changes in protein synthesis in the embryo after NTP treatment. Some research on gene expression was done using microarray and gene ontology analyses. After NTP treatment of seeds, the expression of several genes changed. Hayashi et al. [26] found the upregulation of genes involved in increasing energy production by photosynthesis by upregulating enzymes such as RuBisCO, increasing carbon fixation and production of the plant hormone auxin. Nakano et al. [27] also noted changes in the synthesis of several proteins, but it appears that there were no mutagenic effects on seed embryos in genes associated with plant growth, as the growth increase was not inherited from the first plant generation. NTP treatment of seeds also resulted in upregulation of the expression of the *SnRK2* and *P5CS* genes and, at the same time, downregulation of the expression of the *LEA1* gene. These genes are associated with drought resistance in plants and might be an indicator that the NTP treatment exerted a certain level of stress on seeds. In another study [46], researchers observed an increased expression in redox homeostasis-related genes and in genes responsible for epigenetic changes in tomato seeds exposed to cold plasma treatment for 10 min. All mentioned reports indicate an important activation of gene regulation after plasma treatment of seeds. However, the question is what triggers are produced by plasma and what signals induce changes in gene and protein expression.

### 2.3. The Effects on Enzyme Activity

Differences in enzyme activities may indicate that the plant has been exposed to some type of stress or change in its natural environment. NTP treatment of seeds could potentially act as a stress agent. To assess this possibility, and to explore the mechanisms of seed response to NTP treatment, researchers examined the activity of different enzyme groups.

For example, malondialdehyde (MDA) is measured as an indicator of membrane lipid peroxidation and membrane damage. Many researchers report that seedlings exposed to NTP treatment exhibit lower concentrations of MDA while showing increased germination and growth. This suggests that NTP exposure of seeds (under appropriate NTP parameters) may reduce membrane damage, possibly by accelerating antioxidant machinery [15,20,40,46].

In the course of evolution, plants have developed an antioxidant system that enables them to combat ROS toxicity and to eliminate its negative effects. Antioxidants have various physiological functions, ranging from converting harmful ROS into less reactive species even if present in relatively low concentrations, to acting as a deactivating agent against oxidation. The antioxidant system comprises both enzymatic and nonenzymatic systems. The enzymatic system consists of superoxide dismutase (SOD), catalase (CAT), ascorbate peroxidase (APX), peroxidase (POD), glutathione reductase (GR), polyphenol oxidase (PPO), among others. Upon deleterious or altered conditions of the plant environment, e.g., extreme oxidative stress, antioxidants scavenge the toxic radicals and help the plants to survive. Under such conditions, the level of antioxidant enzyme activity could increase. However, the increase in antioxidant enzyme activity can not only be correlated with a stressful environment, but also with activation of the energy metabolism during seed germination, which triggers many cellular processes where ROS are actively produced [61].

In many experiments conducted by different researchers, NTP-treated seeds showed an increase in SOD, POD and CAT enzymatic activity [19,20,24,44]. The increased antioxidant enzyme activity was found in a few days-old seedlings. Rahman et al. [19] found no changes in SOD and APX activity in seeds treated with NTP. Increased enzyme activity was found only in older seedlings from seeds treated with NTP, with an increase in SOD activity in the roots and APX activity in the shoots, while there were no detectable changes in the seeds right after the NTP treatment. It appears that the increased antioxidant activity is not the first change in the line of seed response to NTP treatment. It may be a secondary response, but it is not clear what the activation mechanism of increased antioxidant enzyme activity is.

The activity of amylase enzyme secreted by the cells of the aleurone layer of the seed is an important factor in seed germination [62]. Amylase splits the starch reserves into simpler molecules, which are then used for early growth of the seedlings. Seeds exposed to NTP showed a higher increase in the activity of the amylase enzyme after 12 and 24 h of imbibition than untreated imbibed seeds. Together with the increased amylase activity, an increase in soluble sugars, which are a product of amylase degradation of starch was also observed [22]. Chen et al. [12] also observed an increased amylase activity in seedlings of NTP-treated seeds which could be correlated with an increase in germination rate. The effect of NTP was also reflected in the amylase activity of young seedlings. Amylase enzymes are normally activated by plant gibberellin (GA) hormones [63], which indicates that hormones respond to NTP treatment earlier than amylase activity. Another possibility for the activation of amylase activity is by nitrogen species and compounds, more precisely nitric oxide (NO), which may also result from NTP treatment of seeds [13,64].

### 2.4. Morphological and Chemical Changes of the Seed Coat

The effect of NTP on the seeds can be observed directly after the NTP treatment. By using a scanning electron microscope (SEM), many researchers noticed the etching effect on the seed coat. Li et al. [20] investigated the morphology of wheat seed coat. The conspicuous mesh-like structures seen in nontreated seeds were gradually destroyed with prolonged DBD plasma treatment. After 10 min of treatment, the boundary layer of these mesh-like structures on the seed coat was difficult to identify. Besides, noticeable cracks in the seed coat were observed. Similar effects of NTP treatment were observed by many other researchers [18,19,21,25,28,40,46]. Park et al. [28] reported of an uneven etching of barley seeds, indicating an uneven interaction of the NTP with the seed surface. This can be attributed to specific chemical interactions of the NTP with the seed surface and the energy distribution of the NTP. Stolárik et al. [21] also noted the uneven effect of NTP on the surface of pea seeds with abrasion and disruption of the seed surface. During prolonged NTP treatment, cleavages and cracks were observed in the seed surface. This suggests that a prolonged exposure to NTP treatment causes more severe changes in the seed surface morphology. In contrast to these results, Zahoranová et al. [33] did not observe any surface damage to the seed coat and only a slightly softer surface of corn seeds. It is apparent that the altered morphology of the seed coat depends on the initial seed coat morphology and the NTP parameters used.

**Figure 4:**
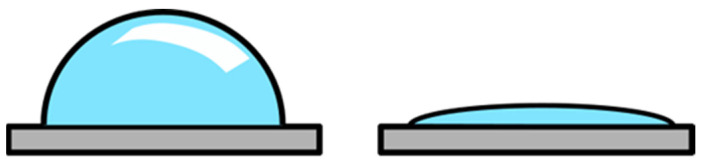
Plasma treatment of seeds decreases water contact angle. Water contact angle of untreated surface on the left, and decreased water contact angle of the surface after plasma pre-treatment on the right.

Numerous experiments showed a decrease in the water contact angle of the seed surface as shown in Figure 4 [22,29,33,49,65,66]. Bormashenko et al. [49] found a decrease in the water contact angle after 15 s treatment with air RF plasma. At the same time, no morphological changes were detected during SEM analysis. This indicates that the change in water contact angle can be attributed to chemical changes that occur during NTP treatment on the seed surface, turning the normally hydrophobic seed coat to a hydrophilic one. Time-of-flight secondary ion mass spectrometry (TOF-SIMS) analysis of seeds treated with NTP demonstrated a 2.5 to 3 times more intense mass peaks corresponding to the presence of oxygen than on untreated seeds. This shows the enrichment of the seed surface with oxygen species by NTP and the introduction of new chemical species on the surface, resulting in a more hydrophilic nature, a reduced water contact angle and an increase in the wettability of the seeds [13,33,34,42,49], which could, to some extent, be attributed to altered surface morphology (nano-structuring of seed coat).

An increase in the hydrophilic properties of biological samples by NTP is an effect similar to the hydrophilization of synthetic polymers, which has been carefully studied in terms of altered surface morphology and functionalization [67,68] as well as influence on the preferential etching of amorphous parts of the polymer [69]. NTP-treatment of polymers introduces new functional groups that have a major impact on the physical and chemical properties of the surface, including its wettability. A common phenomenon accompanying plasma treatment of polymers is also a process called hydrophobic recovery in which polymer surface characteristics revert to their original condition over time and regain hydrophobic properties [70]. In seeds, hydrophobic recovery has not been observed so far [71].

Gómez-Ramírez et al. [13] examined the surface of quinoa seeds with XPS (X-ray photoelectron spectroscopy) and found that the surface was largely affected by the NTP treatment. The exposure of the seeds to DBD plasma for 30 s, or to RF plasma for 10 s, induced a significant increase in the oxygen and nitrogen content of the seed surface at the expense of carbon. They also noted a slight increase in potassium content. Similar effects were observed when monitoring the seed surface with SIMS (secondary-ion mass spectrometry) as reported by Bormashenko et al. [47], confirming the results of the XPS analysis. Prolonged exposures of the seeds to NTP corresponded to an even larger increase in oxygen, nitrogen and potassium content. This indicates the ability of the NTP to oxidize the outermost layers of the seeds due to the presence of highly reactive excited species in air NTP. Similar effects have been described for different seeds exposed to the RF plasma and for some polymers where the depth of plasma-induced oxidation down to 100 nm was found using similar NTP parameters [13]. After exposure of the seeds to water, there was a decrease in potassium and nitrogen species (NO_3_^−^, NO_2_^−^). Gómez-Ramírez et al. [13] suggest that the mechanism behind this is a diffusion of labile potassium and nitrogen species into the interior of the seed after exposure to water. The diffusion of these molecules promotes seed germination in a similar way the exposure to nitrate-rich water influences seed metabolism and water uptake. Zahoranova et al. [33] reported that there were no detectable morphological changes as observed by SEM after NTP treatment of maize seeds. Attenuated total reflection-Fourier-transform infrared spectroscopy (ATR-FTIR) measurements, however, indicated some changes in the chemical groups of plasma-treated seeds, with a decrease in the lipid group and an increase in polar groups containing oxygen and nitrogen. All these results confirm that NTP reduces the hydrophobic surface of the seeds and transforms it into more hydrophilic one.

### 2.5. Plant Hormone Balance

Plant hormones, such as auxin, cytokinin, ethylene, gibberellins (GA), abscisic acid (ABA) and brassinosteroids, are molecules that control various physiological and biochemical processes in the plant. Plant hormones can be associated with germination, growth and development, flowering, fruiting, fruit ripening, dormancy and resistance to various abiotic and biotic stresses [64]. During germination, numerous processes are activated in the seeds, such as the release of stored reserves, activation of metabolism and growth of meristems. During germination and the transition from seed to seedling, plants are very sensitive to environmental factors such as light, temperature and water availability. The reaction to these abiotic factors is often mediated by hormones [63].

Researchers indicate that the treatment of seeds with NTP influences the presence and synthesis of hormones in seeds and seedlings [20,21,28,38,40,41]. The effects depend not only on the concentration of a specific hormone but also on the relationship of the specific hormone to other hormones and signaling molecules. By generating low-pressure NTP, seeds are placed in vacuum that could potentially affect their properties and seed germination. Although according to Mildažienė et al. [38], there was no difference in germination and seedling growth between seeds exposed to vacuum and the untreated seeds, there was a noticeable change in the auxin/cytokinin ratio. This indicates the response of the plant to an external factor (vacuum) by activation of signaling pathways related to auxin and cytokinin phytohormones. Additional treatment with RF NTP caused an increase in gibberellin GA_3_ concentration, while exposure to an RF electromagnetic field reduced the concentration of ABA. Although seeds in the dehydrated state have a high resistance to abiotic stress, they are still able to respond rapidly to short NTP, electromagnetic field or vacuum treatments by producing plant hormones. Ji et al. [14] also noted an increase in GA_3_ concentration in wheat seeds and suggested that hormones and increased activity of hydrolytic enzymes activate seed germination after NTP treatment. Stolárik et al. [21] noted that in NTP-treated seeds, the biosynthesis of auxins and cytokinin, as well as their catabolites and conjugates, was upregulated. Guo et al. [40] reported an increase in ABA content in four-day old wheat seedlings exposed to NTP treatment prior to imbibition. ABA is an important signaling factor in response to dehydration, with the ability to regulate the water status of plants through changing stomatal conductivity and inducing genes involved in dehydration resistance.

There is increasing evidence that the pretreatment of seeds with NTP has an important impact on the hormonal balance. Whether hormones play a fundamental role in the response of seedlings to NTP treatment of seeds, is still not clear and requires additional attention and research.

### 2.6. Germination and Seedling Growth Parameters

Many researchers noted effects of seed exposure to NTP on germination, and later on the growth and development of the seedlings. In many cases, NTP treatment of seeds could increase the germination rate and induce faster germination in different crops [15,19,22,25,29,31,33,40,42,46]. Nevertheless, in numerous other cases, there was no improvement in the germination rate. The differences between the control group and the NTP treated seeds appeared later as improved root and shoot growth, or root branching of the seedlings [32,33,39,41,72,73], indicating various outcomes dependent on the type of plasma, treatment conditions, plant species and even the plant variety. Thus, more systematic studies correlating treatment conditions with seed types and varieties should be performed. In particular, it is recommended to examine details about the discharge parameters, in particular the concentration of gaseous impurities which may significantly influence the plasma parameters [74].

### 2.7. Resistance to Stress

Seeds treated with NTP exhibit interesting responses when exposed to abiotic stresses such as drought and salinity. Guo et al. [40] simulated polyethylene glycol (PEG)–induced drought stress on wheat seeds. PEG-induced drought stress caused significant oxidative damage to germinating wheat seedlings, which was confirmed by increased H_2_O_2_ and O_2_ levels, higher MDA concentration and increased proline and soluble sugar content. In contrast, nonthermal DBD plasma treatment mitigated the oxidative damage of drought stress in wheat seeds through the induced expression of the functional gene *LEA1* and regulatory genes such as *SnRK2* and *P5CS*, increasing ABA levels and accelerating the enzymatic activity of antioxidants. The MDA content associated with lipid peroxidation and membrane damage decreased in seeds pretreated with NTP and exposed to drought stress compared to drought-exposed seeds without NTP treatment. Ling et al. [15] came to similar results for NTP DBD plasma treatment of oilseed rape seeds, where statistically significant differences were found between two varieties of oilseed rape. Bafoil et al. [25] conducted experiments on how salinity stress and NTP treatment affect germination of *Arabidopsis thaliana* seeds. Although the germination rate of NTP-treated seeds increased, NTP treatment could, to a certain extent, compensate for the negative effect of salinity stress. Based on the above results, NTP treatment of seeds has a high potential in regions where crop plants are frequently exposed to drought. Furthermore, the initial water uptake of NTP-treated seeds is definitely increased as shown by numerous authors [12,33,48,73,75].

Recently researchers have also investigated how NTP could alleviate the stress induced by potentially toxic metals (Zn) and metal oxide (ZnO) nanoparticles during seed germination and plant development. Iranbakhsh et al. [30] reported that the treatment of seeds with NTP had a great growth-promoting and protective effect. ZnO nanoparticles were proposed to have a less toxic effect, as growth and differentiation of NTP-treated seeds were improved. Promotion of phenylalanine ammonia lyase (PAL) and peroxidase activity after cold plasma treatment of seeds could give insight to the plant’s resistance to abiotic stress, caused by plasma compounds such as NO, ozone and UV radiation. Increased activity of PAL and peroxidase enzymes by plasma pretreatment of seeds could thus act as a protective mechanism of the seedlings, involved in the alleviation of toxic effects caused by nano-ZnO. This is an important new aspect of NTP influence on seeds and plants with great potential in the field of bioremediation.

### 2.8. Transfer of the Traits Induced by Nonthermal Plasma to the Next Generation

As has been demonstrated, NTP treatment affects the seeds and their properties. It is important to know whether the effects have an impact on seeds and plants of future generations. Hayashi et al. [26] reported that the growth of second-generation plants was similar to that of seeds not treated with NTP, indicating that no mutation of growth-related genes occurred during the NTP treatment of first-generation seeds. Gene expression analysis of *Arabidopsis thaliana* seeds, however, showed a different gene expression pattern in the second-generation compared to the first-generation seeds. This confirms that no mutation, but an epigenetic effect occurred during the enhanced germination of the first-generation seeds. In view of these results, it is still unclear whether NTP causes changes in seeds that are passed on to future generations. It is necessary to conduct additional experiments on the possibility that NTP has an effect on the inheritance of NTP-induced traits to next-generation seeds. Due to time consuming experiments, not much work has been conducted in this direction.

## 3. Conclusions

NTP technology has great potential in agriculture as a new technology for seed priming. It is an ecological, environmentally friendly and rather simple technology. Understanding the mechanisms behind its effects on improved seed germination, plant growth, improved yield and stress tolerance is critical for future exploitation of its benefits and applications. Many researchers have focused on different levels of NTP effects on seeds, ranging from chemical and morphological changes on the seed surface to changes in water contact angle, water absorption, DNA (de)methylation, gene expression patterns, potential DNA damage, the speed and rate of germination, the growth of the seedlings, the activity of various enzymes, the presence of hormones, the transfer of improved properties to the next generation, plant resistance to drought stress and even metal toxicity. However, much more work, focused on systematic studies on the influence of various types of NTP and different crops as well as crop variety, should be conducted. As was already shown, the types of NTP (RF, APPJ, MW, etc.) affects seeds and seedlings, but it is also important to emphasize that researchers use different NTP experimental setups, which may also influence seed modification. Thus, in many cases, controversial results are reported in the literature, and it is hard to provide a general rule on which treatment conditions have the most influence on seeds in terms of their surface properties as well as biological response. Here, it is worth mentioning that the discharge itself does not interact with the seeds, but rather the reactive gaseous species and UV/VUV radiation produced in NTP sustained by the discharge. From this point of view, the scientific challenge remains appropriate plasma characterization, which may not be trivial, in particular for atmospheric-pressure plasma jets where huge gradients in the density of reactive species are common.

The main mechanisms of changes occurring during and after NTP treatment of seeds are still unknown. It is also important to assess which NTP properties are responsible for the changes. It is suggested that oxygen and nitrogen reactive species play a major role in NTP priming of seeds. However, the presence of UV and/or VUV radiation, electromagnetic field, possible temperature fluctuations and other products and by-products of NTP generation can also contribute to changes in seeds. The response of seeds to NTP could additionally be species and variety-dependent. Furthermore, the size of seeds of the same species could also play a decisive role, as the distribution of NTP energy on the seed surface could be different.

## Figures and Tables

**Table 1:** An overview of experimental cold plasma parameters and their effects on different seeds. DBD: dielectric. DCSBD: diffuse coplanar surface barrier discharge. RF-CCP: radio frequency capacitively coupled plasma. RF-EMF: radio frequency electro-magnetic field. LPDBD: low pressure dielectric barrier discharge.

Seed Type	Plasma Parameters	Exposure Time	Result Summary	Ref.
*Arabidopsis thaliana*	DBD, 10 kHz, 10 kV, atmospheric pressure, air	15 min	Plasma pretreated seeds germinated faster, but the final germination rate was not significantly increased; germination was improved under salinity conditions as germination decrease caused by salinity stress was partially restored.	[25]
*Arabidopsis thaliana, Raphanus sativus*	RF, 13,56 MHz, 60 W, 20–80 Pa, O_2_		No significant impact on plant growth, but the gene expression patterns were changed, also when comparing the first and the second generation of seeds.	[26]
*Arabidopsis thaliana, Raphanus sativus*	RF, 13.56 MHz, 60 W, 20–80 Pa, Ar, O_2_	5, 15, 30 and 60 min	An enhanced seedling growth (at 80 Pa for 10 or 20 min) and changed gene expression pattern; growth enhancement was not inherited by the second-generation plants.	[27]
Barley (*Hordeum vulgare*)	SDBD (AC), 30 kHz, 400 W, atmospheric pressure, N_2_ and air	10, 20, 40 and 80 s	Denser and longer roots and higher shoots in plasma-exposed seedlings; an increase in GABA levels.	[28]
Beans *(Phaseolus vulgaris*)	RF, 10 MHz, 20 W, 6.7 × 10^−2^ Pa, air	2 min	Faster initial germination, but the final germination rate was the same; hydrophilization of the seed coat surface and accelerated water uptake.	[29]
Bell pepper (*Capsicum annuum*)	DBD (AC), 23 kHz, 11 kV, 80 W, atmospheric pressure, Ar	60, 120 s	Growth-promoting and protecting effects on seedlings; inhibition and delay of nano ZnO toxicity and its negative effects on plant growth and differentiation.	[30]
Brown rice	DC, 1–3 kV, 800 Pa, air	10 min	Increased germination rate, seedling length, water uptake, and GABA levels with optimum treatment at 3kV for 10 min.	[12]
Chickpea (*Cicer arietinum*)	surface microdischarge (SMD), complex 20-ms cycle, atmospheric pressure, air	0.5–5 min	A noticeable increase in germination rate and root and shoot length.	[31]
Maize (*Zea mays*)	DCSBD, 14 kHz, 370 W, atmospheric pressure, air	60, 120 s	No change in the germination rate, noticeably longer roots and bigger wet and dry biomass of plants after plasma treatment.	[32]
Maize (*Zea mays*)	DCSBD, 14 kHz, 20 kV, 400 W, atmospheric pressure, air	30–300 s	No statistically significant changes in the germination rate; at longer exposure times, a decrease in germination rate; an increase in root length and shoot height.	[33]
Mung beans (*Vigna radiata*)	RF-CCP, 13.56 MHz, 40 and 60 W, 20 Pa, air	10, 15 and 20 min	Faster germination, an increased germination rate, higher shoots, an increase in water-soluble sugars and higher amylase and phytase activity.	[22]
Mung beans (*Vigna radiata*)	microplasma, 9 kHz, 0–20 kV, 25 W, atmospheric pressure, N_2_, He, air and O_2_	10 min	Different effects depending on feed gas, higher germination rate in air and helium plasma, an increase in plant height for air plasma, no significant effects on seeds in O_2_ plasma.	[34]
Pea (*Pisum sativum*)	DBD, 14 kHz, 20 kV, 400 W, atmospheric pressure, air/N_2_/O_2_/N_2_ and O_2_	60, 180 and 300 s	More DNA damage than in nontreated samples; an ambient air plasma had the least damaging effects on seed DNA, compared to plasma treatment with different mixtures of O_2_ and N_2_.	[35]
Pea (*Pisum sativum*)	DCSBD, 14 kHz, 20 kV, atmospheric pressure, air	60–300 s	No increase in DNA damage. After the application of DNA-damage agent Zeocin, an increase in DNA damage. The plasma-treated seeds had a lower level of DNA damage than untreated seeds.	[36]
Pea (*Pisum sativum*)	DCSBD, 14 kHz, 10 kV, 370 W, atmospheric pressure, air	60–600 s	Increased biosynthesis of auxin and cytokinins as well as their catabolites and conjugates; a noticeable increase in germination rate and root and shoot length.	[21]
Peanut (*Arachis hypogaea*)	RF-CCP, 13.56 MHz, 60–140 W, 150 Pa, He	15 s	Improved germination rate and peanut yield after plasma treatment of seeds at 120 W.	[37]
Quinoa (*Chenpodium quinoa*)	RF and DBD, 1 kHz, 8.2 kV, 6.4 W, 500 and 0.1 mbar, air	10, 30, 60, 180 and 900 s	A higher germination rate in seeds treated for 10 s with RF plasma or 180 or 900 s by DBD plasma. A drastically changed chemistry of the seed coat outer layer	[13]
Rapeseed (*Brassica napus*)	RF-CCP, 13.56 MHz, 100 W, 150 Pa, He	15 s	Increased germination rate and shoot and root growth. Germination rate, germination index and vigor of seeds exposed to drought stress and treated with plasma was increased, along with plant growth and a decrease in MDA content compared to untreated seeds exposed to drought	[15]
Sunflower (*Helianthus annuus*)	RF-EMF, 5.28 MHz, atmospheric pressure, air	5, 10 and 15 min	No significant changes in the germination rate of plasma and EMF treated seeds; noticeable changes in phytohormone balance; after short exposure of seeds to RF-CP or RF-EMF a long-term effect on gene expression in leaves, mostly stimulating expression of proteins involved in photosynthesis and its regulation.	[38]
	RF-CP, 200 Pa, air	2, 5 or 7 min		
Soybean (*Glycine max*)	60 kHz, 10.8–22.1 kV, 3.4–15.6 W, atmospheric pressure, Ar	12, 24, 48, 60, 120 and 180 s	A slightly higher germination rate and enhanced root and shoot growth; changes in DNA methylation level, an increased SOD, POD and CAT enzyme activity.	[24]
Wheat (*Triticum aestivum*)	SDBD (AC), 50 Hz, atmospheric pressure, air	5, 15 and 20 min	The germination rate unchanged; an enhanced root growth (especially in seedlings treated with plasma for 15 min).	[39]
Wheat (*Triticum aestivum*)	DBD, 50 Hz, 13 kV, atmospheric pressure, air	4 min	Increased germination rate, root length and shoot height; noticeable changes in the expression of genes *LEA1*, *SnRK2* and *P5CS*; increased proline and soluble sugar levels in normal water conditions and in seedlings exposed to drought conditions. A decrease in MDA content in seeds under drought stress. An increase in SOD, POD and CAT enzyme activity.	[40]
Wheat (*Triticum aestivum*)	RF, 3 × 10^9^ MHz, 60, 80 and 100 W, 150 Pa, He	1–5 shots of high voltage nanosecond	No changes in the germination rate and speed; enhanced root and shoot growth and an increased yield.	[41]
Wheat (*Triticum aestivum*)	DBD (AC), 50 kHz, 13 kV, 1.5 W, atmospheric pressure, air	1, 4, 7, 10 and 13 min	Increased root and shoot growth, an increase in proline and soluble sugar levels. With increased exposure time a noticeable decline of MDA content and an increase in SOD and POD enzyme activity; increased germination rate.	[20]
Wheat (*Triticum aestivum*)	DBD, 50 kHz, 80 kV, atmospheric pressure, air	30, 60 and 80 s	Improved seed germination rate and an increase in root growth. Shoots longer only in seeds treated with plasma for 30 s and no retention time, and seeds treated with plasma for 60 s and retention time of 24 h.	[42]
Wheat (*Triticum aestivum*)	DBD (AC), 50 kHz, 13 kV, atmospheric pressure, air, O_2_, N_2_, Ar		In seeds, treated with air, N_2_ or Ar plasma an increase in root length; no increase in seeds treated with O_2_ plasma.	[18]
Wheat (*Triticum aestivum*)	LPDBD, 5 kHz, 4.5 kV, 45 W, 10 torr, Ar/O_2_ or Ar/air	90 s	Shorter roots and longer shoots compared to the control seedlings; an elevated SOD enzymatic activity in roots and a higher level of H_2_O_2_ in roots and shoots in seedlings treated with O_2_/Ar plasma; with Ar/air plasma an increase in CAT activity in leaves.	[19]
Wheat (*Triticum aestivum*)	RF, 13.56 MHz, 80 W, 0.1 mbar, air	60, 120, 180 and 240 s	With Plasma treatment for 180 s a higher wheat yield and increased plant photosynthesis.	[43]
Maize, wheat, soybeans, tobacco	RF-CCP, 13.56 MHz, 50–1000 W, 30–200 Pa, air	5–90 s	An increase in the germination rate of maize and seedling growth of wheat; SOD and POD enzymatic activity also increased; an increase in the yield of tobacco (20%) and soybean (4%).	[44]
